# Poisoning with central stimulant drugs: an observational study from Oslo, Norway

**DOI:** 10.1186/s12245-022-00457-x

**Published:** 2022-09-29

**Authors:** Erlend Ingebrigtsen, Per Sverre Persett, Mette Brekke, Fridtjof Heyerdahl, Knut Erik Hovda, Odd Martin Vallersnes

**Affiliations:** 1 Faculty of Medicine, University of Oslo, Oslo, Norway; 2 Department of Surgery, Orkdal Hospital, St Olav’s Hospital, Orkdal, Norway; 3 Department of Acute Medicine, Oslo University Hospital, Oslo, Norway; 4 General Practice Research Unit, University of Oslo, Oslo, Norway; 5 Air Ambulance Department, Oslo University Hospital, Oslo, Norway; 6 The Norwegian Air Ambulance Foundation, Oslo, Norway; 7 Institute of Clinical Medicine, University of Oslo, Oslo, Norway; 8 Department of Acute Medicine, The Norwegian CBRNe Centre of Medicine, Oslo University Hospital, Oslo, Norway; 9 Department of General Practice, University of Oslo, Oslo, Norway; 10 Department of Emergency General Practice, Oslo Accident and Emergency Outpatient Clinic, City of Oslo Health Agency, Oslo, Norway

**Keywords:** Poisoning, Toxicity, Central stimulant drugs, Amphetamine, Methamphetamine, Cocaine, MDMA, NPS, Ecstasy, Recreational drugs

## Abstract

**Background:**

The use of central stimulant drugs causes significant morbidity. We describe poisonings with central stimulant drugs and compare the different central stimulants concerning combinations with other drugs, treatment, and clinical course.

**Methods:**

Patients presenting from 1 October 2013 to 31 March 2016 with poisoning related to the recreational use of central stimulant drugs were retrospectively included at a primary care emergency outpatient clinic and at a hospital emergency department in Oslo, Norway. Diagnosis of toxic agents was mainly based on the clinical assessment of the doctor treating the patient. Amphetamine and methamphetamine were co-categorized as amphetamine.

**Results:**

Among the 1131 cases of acute poisoning with central stimulant drugs at the outpatient clinic, amphetamine was involved in 808 (71.4%), cocaine in 252 (22.3%) methylenedioxymethamphetamine (MDMA) in 104 (9.2%), and methylphenidate in 13 (1.1%). Among the 211 cases at the hospital, amphetamine was involved in 167 (79.1%), cocaine in 60 (28.4%), and MDMA in 38 (18.0%). Amphetamine was frequently combined with opioids (40.1% at the outpatient clinic and 41.9% at the hospital) and benzodiazepines (28.3% and 45.5%), while MDMA often was combined with ethanol (64.4% and 71.1%), as was cocaine (62.7% and 61.7%). Sedation was given in 5.2% and 38.4% of cases, naloxone in 9.4% and 37.0%, and flumazenil in 0.1% and 28.0%. In total, 16.5% of the cases at the outpatient clinic were transferred to a hospital for medical review and 8.5% to a psychiatric hospital. Among the hospital patients, 92.9% were admitted to intensive care.

**Conclusion:**

Amphetamine was the most common central stimulant drug involved in acute poisoning in Oslo, often combined with opioids and benzodiazepines.

## Background

Central stimulant use is a global problem [1]. The use of amphetamines has increased in North America, to a last-year use prevalence of 3.9% in the population aged 15–64 years [1], along with an increasing number of overdose deaths involving central stimulants [2]. Cocaine use is also widespread, with a last-year use prevalence of 2.7% in Oceania and 1.6% in South America, as is the use of ecstasy (mainly 3,4-methylenedioxymethamphetamine (MDMA)) in Oceania with a last-year use prevalence of 2.2% [1]. The last-year use prevalence of central stimulants is markedly lower in Asia and Africa, in the range of 0.1–0.4% [1].

The central stimulant drugs cocaine, amphetamine, and methamphetamine are, after cannabis, the most frequently used illegal drugs in Norway [1, 3, 4]. Cocaine use has a last-year prevalence in Norway of 0.8% in the age group 15–64 years, close to the European mean at 1.0% [1]. The last-year prevalence of amphetamine/methamphetamine use is 0.6%, equal to the European mean, but in wastewater analyses in the Norwegian capital city, Oslo, larger amounts of amphetamines are found than in other European cities [1, 4]. Increasing amounts of cocaine are also found in wastewater analyses in Oslo [4]. The last-year prevalence of the use of ecstasy in Norway is 0.9%, again close to the European mean at 0.7% [1].

During the last two decades, several hundred new drugs have appeared in Europe, often termed new psychoactive substances (NPS) or designer drugs, among them several hundred with stimulant effects [5, 6]. Police seizures confirm this trend also in Norway [3].

The incidence of drug overdose death is high in Norway, 67.8 per million inhabitants aged 15–64 years per year vs. 18.3 in Europe in total [4]. Opioids are responsible for about 80% of overdose deaths in Norway [7]. During the last decade, central stimulant drugs have been responsible for just above 5% [7].

The toxicity of central stimulants is related to their sympathomimetic effects, including agitation, tachycardia, arrhythmias, hypertension, acute coronary syndrome, stroke, hyperthermia, hallucinations, and psychosis [6, 8, 9]. As the range of available drugs and the pattern of stimulant drug use change, updated studies are needed to keep track of trends and consequences for acute poisoning.

We describe patients treated for acute poisoning with central stimulant drugs in Oslo, Norway. We compare cases involving amphetamines, cocaine, MDMA, and other stimulants concerning demographics, combinations with other drugs, clinical features, treatment, and clinical course.

## Methods

### Design

The study was observational with retrospective data registration from the electronic patient records at the Oslo Accident and Emergency Outpatient Clinic (OAEOC) and at Oslo University Hospital (OUH) from 1 October 2013 to 31 March 2016.

### Settings

Oslo is the capital city of Norway and had a population of 658,390 as per 1 January 2016 [10]. About one million live in the Oslo metropolitan area. The OAEOC is a primary care emergency outpatient clinic, serving the entire city at all hours. There are about 200,000 consultations per year. The Norwegian emergency health care system is two-tiered with a gate-keeping function. Unless triaged for hospital care by the ambulance service, patients are seen in primary care and cannot present directly to a hospital emergency department (ED). Concerning poisoning with alcohol and/or recreational drugs, the OAEOC functions as a pre-ED for the city hospitals, managing the less complicated cases with limited diagnostic and treatment resources [11]. The OUH is one of four hospitals in Oslo, with both primary and tertiary referral functions. About 4600 patients are seen in the OUH ED per year.

Data from the two centers were analyzed separately. As some patients were transferred from the OAEOC to the OUH, the patient populations have some overlap.

### Participants

Using the case definition developed by the European Drug Emergencies Network (Euro-DEN) [12], we registered all patients with acute toxic effects related to recreational drug use presenting at the two centers. Cases involving only alcohol were not included, nor poisonings related to self-harm, suicide attempts, inflicted by others, or accidental exposures. Eligible cases were found by searching the patient registration lists in the electronic patient records.

From this material, we extracted all cases involving at least one central stimulant drug. From the OAEOC total of 3733 cases, 1131 (30.3%) were included. From the OUH total of 457 cases, 211 (46.2%) were included.

### Data collection and classification

We collected data from the electronic patient records at both centers and from paper observational charts at the OAEOC, using the Euro-DEN variable set [12]. We registered age, gender, time of presentation, time of discharge, whether the patient was brought by ambulance, disposition from the ED, death in hospital, toxic agents taken, clinical observations at presentation (cardiac arrest, level of consciousness measured by Glasgow Coma Scale (GCS) score, respiratory rate, heart rate, blood pressure, temperature, serum lactate (hospital only)), clinical features of the poisoning episode (hyperthermia (≥ 39 °C), vomiting, headache, anxiety, hallucinations, agitation, psychosis, seizures, palpitations, chest pain, hypertension (≥ 180 mmHg), hypotension (≤ 90 mmHg), arrhythmias), and treatment (intubation, sedation, naloxone, flumazenil, any treatment beyond mere observation).

Classification of toxic agents was based on the clinical diagnosis made by the doctor treating the patient as entered in the patient records, in its turn based on all available information, i.e., information from the patient and companions, and on the clinical picture. The term “unspecified stimulant” was used in the patient records when the patient reported taking a stimulant but did not know which specific drug or when the clinical picture was consistent with a stimulant toxidrome and no information on specific drugs was available.

At the OUH, the clinical diagnosis was supplemented by toxicological analyses in urine samples in 124 (58.8%) cases. For gamma-hydroxybutyrate (GHB), gas chromatography–flame ionization detector (GC-FID) was used until 9 October 2014, when replaced by gas chromatography–mass spectrometry (GC–MS). For other agents, immunological screening was followed by liquid chromatography–tandem mass spectrometry (LC–MS/MS) confirmation.

We co-categorized amphetamine and methamphetamine as amphetamine. Z-drugs were categorized as benzodiazepines.

When converting continuous variables into categorical variables (tachypnoea (respiratory rate ≥ 20/min), bradypnoea (respiratory rate < 10/min), tachycardia (heart rate ≥ 100/min), bradycardia (heart rate < 50/min), and hyperlactataemia (serum lactate ≥ 3.00 mmol/L)) missing values were treated as the absence of the relevant clinical feature. Commonly used clinical thresholds were used when defining the categorical variables.

For comparisons, we grouped the cases as follows: amphetamine, cocaine, MDMA, other stimulants (all other specified or unspecified stimulants), and multiple stimulants (all cases involving more than one stimulant drug).

### Statistical analyses

Analyses were done in IBM SPSS version 26, 27, and 28. We described the data using proportions for categorical variables and medians and interquartile ranges for continuous variables. The chi-square test was used when comparing categorical variables. When chi-square test assumptions were not met as more than 20% of the cells had an expected value of less than 5, we used Fisher’s exact test instead. The Kruskal–Wallis test was used when comparing continuous variables. The level of significance was set at *p* < 0.05.

### Ethical approval and consent to participate

The study was done as part of a quality improvement study, as per the Norwegian Law on Health Personnel §26. The OUH Information Security and Privacy Office (ref no 2013/3706) assessed the study to be a quality improvement study. Hence, the need for approval by an ethics committee was waived by the Norwegian ethics committee regulations for quality improvement studies. The need for informed consent from the patients was also waived by the Norwegian ethics committee regulations for quality improvement studies. Data were registered anonymously from electronic medical records. The study was performed in accordance with guidelines and regulations.

## Results

Among 1131 included cases at the outpatient clinic, 862 (76.2%) were males and the median age was 30 years (interquartile range (IQR) 25–38) (Table 1). Amphetamine was involved in 808 (71.4%) cases, cocaine in 252 (22.3%), MDMA in 104 (9.2%), unspecified stimulants in 24 (2.1%), methylphenidate in 13 (1.1%), stimulant NPS in 4 (0.3%), and other stimulants in 4 (0.3%).

**Table 1:** Central stimulant drug poisoning – outpatient clinic patients. Demographics, drugs combined, treatment, and disposition

	**Amphetamine *n* (%)**	**Cocaine *n* (%)**	**MDMA *n* (%)**	**Other *n* (%)**	**Multiple *n* (%)**	**Total *n* (%)**	***p* value**
**Males**	555 (74.2)	167 (85.2)	51 (71.8)	32 (80.0)	57 (75.0)	862 (76.2)	0.021
**Age**^a,b,c^	32 (26–41)	28 (24–32)	26 (23–30)	28 (23–39)	28 (24–34)	30 (25–38)	< 0.001
**Drugs in combination**^d^							
*Ethanol*	138 (18.4)	128 (65.3)	50 (70.4)	13 (32.5)	36 (47.4)	365 (32.3)	< 0.001
*Opioids*^e^	310 (41.4)	25 (12.8)	7 (9.9)	5 (12.5)	15 (19.7)	362 (32.0)	< 0.001
*Benzodiazepines*	216 (28.9)	24 (12.2)	14 (19.7)	3 (7.5)	17 (22.4)	274 (24.2)	< 0.001
*GHB*	69 (9.2)	11 (5.6)	4 (5.6)	1 (2.5)	11 (14.5)	96 (8.5)	0.069
*Cannabis*	78 (10.4)	31 (15.8)	8 (11.3)	4 (10.0)	7 (9.2)	128 (11.3)	0.29
*Other/unknown*	23 (3.1)	9 (4.6)	-	3 (7.5)	3 (3.9)	38 (3.4)	0.15
**Brought by ambulance**	373 (49.9)	102 (52.0)	33 (46.5)	17 (42.5)	39 (51.3)	564 (49.9)	0.80
**Treatment**^f^	150 (20.1)	38 (19.4)	8 (11.3)	5 (12.5)	12 (15.8)	213 (18.8)	0.29
**Sedation**	31 (4.1)	16 (8.2)	5 (7.0)	1 (2.5)	6 (7.9)	59 (5.2)	0.12
**Naloxone**	93 (12.4)	5 (2.6)	2 (2.8)	3 (7.5)	3 (3.9)	106 (9.4)	< 0.001
**Flumazenil**	1 (0.1)	-	-	-	-	1 (0.1)	1.00
**Length of stay**^a,g^	3:24 (1:44–5:25)	2:24 (1:30–4:28)	2:20 (1:32–5:07)	1:59 (1:09–3:23)	2:40 (1:35–5:23)	3:01 (1:38–5:12)	< 0.001
**Disposition**							< 0.001
*Hospital, medical ED*	121 (16.2)	31 (15.8)	9 (12.7)	10 (25.0)	16 (21.1)	187 (16.5)	
*Psychiatric hospital*	65 (8.7)	10 (5.1)	3 (4.2)	14 (35.0)	4 (5.3)	96 (8.5)	
*Medical discharge*	451 (60.3)	121 (61.7)	46 (64.8)	10 (25.0)	43 (56.6)	671 (59.3)	
*Self-discharge*	111 (14.8)	34 (17.3)	13 (18.3)	6 (15.0)	13 (17.1)	177 (15.6)	
**Total**	748 (100)	196 (100)	71 (100)	40 (100)	76 (100)	1131 (100)	

*ED* Emergency department, *GHB* Gammahydroxybutyrate, *MDMA* Methylenedioxymethamphetamine

^a^Median (interquartile range)

^b^Missing: 4 (amphetamine group)

^c^Total age range 15–66 years

^d^As more than one additional drug may have been taken, totals may add up to more than 100%

^e^Heroin was involved in 310/362 (85.6%) of the opioid cases

^f^Any treatment beyond mere observation. No patients were intubated

^g^Total range length of stay 0:08–36:53 h

Among 211 included cases at the hospital, 153 (72.5%) were males and the median age was 31 years (IQR 25–38) (Table 2). Amphetamine was involved in 167 (79.1%) cases, cocaine in 60 (28.4%), MDMA in 38 (18.0%), stimulant NPS in 4 (1.9%), and other stimulants in 2 (0.9%).

**Table 2:** Central stimulant drug poisoning – hospital ED patients. Demographics, drugs combined, treatment, and disposition

	**Amphetamine *n* (%)**	**Cocaine *n* (%)**	**MDMA *n* (%)**	**Multiple *n* (%)**	**Total *n* (%)**	***p* value**
**Males**	86 (71.1)	27 (84.4)	6 (75.0)	34 (68.0)	153 (72.5)	0.40
**Age**^a,b,c^	33 (28–40)	28 (23–36)	26 (24–30)	28 (24–35)	31 (25–38)	0.013
**Drugs in combination**^d^						
*Ethanol*	44 (36.4)	20 (62.5)	8 (100)	31 (62.0)	103 (48.8)	< 0.001
*Opioids*^*e*^	50 (41.3)	7 (21.9)	1 (12.5)	20 (40.0)	78 (37.0)	0.095
*Benzodiazepines*	55 (45.5)	4 (12.5)	-	22 (44.0)	81 (38.4)	< 0.001
*GHB*	63 (52.1)	4 (12.5)	2 (25.0)	20 (40.0)	89 (42.2)	< 0.001
*Cannabis*	34 (28.1)	6 (18.8)	3 (37.5)	26 (52.0)	69 (32.7)	0.006
*Other/unknown*	6 (5.0)	6 (18.8)	1 (12.5)	6 (12.0)	19 (9.0)	0.049
**Brought by ambulance**	114 (94.2)	28 (87.5)	7 (87.5)	49 (98.0)	198 (93.8)	0.16
**Treatment**^f^	101 (83.5)	25 (78.1)	7 (87.5)	48 (96.0)	181 (85.8)	0.055
**Intubated**	17 (14.0)	7 (21.9)	1 (12.5)	21 (42.0)	46 (21.8)	0.001
**Sedation**	29 (24.0)	16 (50.0)	3 (37.5)	33 (66.0)	81 (38.4)	< 0.001
**Naloxone**	51 (42.1)	8 (25.0)	-	19 (38.0)	78 (37.0)	0.044
**Flumazenil**	40 (33.1)	5 (15.6)	-	14 (28.0)	59 (28.0)	0.069
**Length of stay**^a,g^	12:06 (6:09–29:26)	26:04 (11:03–57:18)	12:00 (7:59–16:49)	17:43 (12:48–47:32)	14:19 (8:05–33:12)	0.011
**Disposition from ED**						0.77
*Intensive care unit*	111 (91.7)	29 (90.6)	8 (100.0)	48 (96.0)	196 (92.9)	
*Psychiatric ward*	1 (0.8)	-	-	1 (2.0)	2 (0.9)	
*Other hospital ward*	6 (5.0)	2 (6.3)	-	-	8 (3.8)	
*Medical discharge*	1 (0.8)	-	-	-	1 (0.5)	
*Self-discharge*	2 (1.7)	1 (3.1)	-	1 (2.0)	4 (1.9)	
**Total**	121 (100)	32 (100)	8 (100)	50 (100)	211 (100)	

*ED* Emergency department, *GHB* Gammahydroxybutyrate, *MDMA* Methylenedioxymethamphetamine

^a^Median (interquartile range)

^b^Missing: 3 (amphetamine group 1; cocaine group 2)

^c^Total age range 18–58 years

^d^As more than one additional drug may have been taken, totals may add up to more than 100%

^e^Heroin was involved in 48/78 (61.5%) of the opioid cases

^f^Any treatment beyond mere observation

^g^Total range length of stay 0:35–442:13 h

Amphetamine was frequently combined with opioids, 310/748 (41.4%) cases at the outpatient clinic and 50/121 (41.3%) at the hospital, and with benzodiazepines, 216/748 (28.9%) and 55/121 (45.5%) cases, respectively (Tables 1 and 2). Combining opioids and benzodiazepines was also frequent among patients having taken multiple central stimulant drugs. Then again, amphetamine was involved in most cases in the multiple stimulant group, 60/76 (78.9%) at the outpatient clinic and 46/50 (92.0%) at the hospital. When amphetamine and opioids were combined, benzodiazepines had also been taken in 120/324 (37.0%) of the cases at the outpatient clinic and in 35/70 (50.0%) at the hospital.

**Fig. 1:**
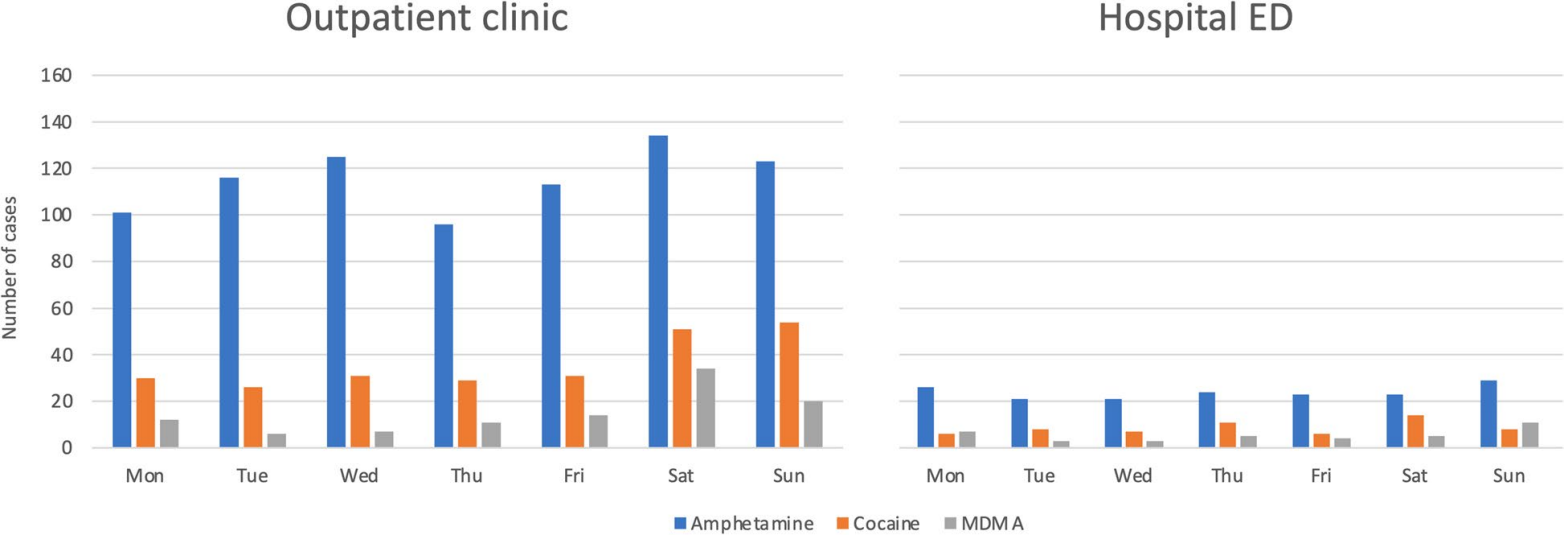
Central stimulant drug poisoning per day of week in Oslo, Norway. Patients treated at an outpatient clinic and in a hospital ED in Oslo, Norway from October 1, 2013, to March 31, 2016 (*n* = 1342). Comparisons across weekdays: Outpatient clinic: amphetamine, *p* = 0.15; cocaine, *p* = 0.001; MDMA, *p* < 0.001; Hospital ED: amphetamine, *p* = 0.92; cocaine, *p* = 0.42; MDMA, *p* = 0.19. ED, emergency department; MDMA, methylenedioxymethamphetamine

Cocaine and MDMA were frequently combined with ethanol, both at the outpatient clinic and at the hospital (Tables 1 and 2). At the outpatient clinic, cocaine and MDMA patients more frequently presented during weekends (Fig. 1).

No patients died at the outpatient clinic. Two patients died at the hospital. Both presented in cardiac arrest and died after 2 days in the intensive care unit. In both cases, amphetamine, cocaine, and opiates were identified in the laboratory analyses.

Tachycardia, reduced GCS score, and agitation were the most common clinical features at the outpatient clinic (Table 3). At the hospital, reduced GCS score, anxiety, and agitation were the most common (Table 4). Palpitations and chest pain were most common in the cocaine group in both settings, as were arrhythmias at the hospital.

**Table 3:** Clinical features of poisoning involving central stimulant drugs – outpatient clinic patients

	**Amphetamine *n* (%)**	**Cocaine *n* (%)**	**MDMA *n* (%)**	**Other *n* (%)**	**Multiple *n* (%)**	**Total *n* (%)**	***p* value**
**Tachypnoea** *(RR* ≥ *20/min)*^a^	120 (16.0)	38 (19.4)	16 (22.5)	7 (17.5)	17 (22.4)	198 (17.5)	0.40
**Bradypnoea** *(RR* < *10/min)*^a^	29 (3.9)	3 (1.5)	-	1 (2.5)	3 (3.9)	36 (3.2)	0.23
**Tachycardia** *(HR* ≥ *100/min)*^a^	298 (39.8)	98 (50.0)	42 (59.2)	20 (50.0)	41 (53.9)	499 (44.1)	0.001
**Bradycardia** *(HR* < *50/min)*^a^	9 (1.2)	1 (0.5)	1 (1.4)	-	-	11 (1.0)	0.80
**Hypertension** *(SBP* ≥ *180 mmHg)*	2 (0.3)	1 (0.5)	1 (1.4)	-	-	4 (0.4)	0.32
**Hypotension** *(SBP* ≤ *90 mmHg)*	21 (2.8)	3 (1.5)	1 (1.4)	1 (2.5)	1 (1.3)	27 (2.4)	0.85
**Hyperthermia** *(tp* ≥ *39.0 °C)*	13 (1.7)	-	-	-	-	13 (1.1)	0.28
**Level of consciousness**^a,b^							0.83
*GCS score 15*	417 (55.9)	119 (60.7)	38 (53.5)	22 (57.9)	46 (60.5)	642 (57.0)	
*GCS score 8–14*	316 (42.4)	76 (38.8)	33 (46.5)	16 (42.1)	29 (38.2)	470 (41.7)	
*GCS score* ≤ *7*	13 (1.7)	1 (0.5)	-	-	1 (1.3)	15 (1.3)	
**Vomiting**	21 (2.8)	15 (7.7)	9 (12.7)	2 (5.0)	6 (7.9)	53 (4.7)	< 0.001
**Headache**	23 (3.1)	8 (4.1)	3 (4.2)	1 (2.5)	5 (6.6)	40 (3.5)	0.48
**Anxiety**	91 (12.2)	45 (23.0)	8 (11.3)	1 (2.5)	15 (19.7)	160 (14.1)	< 0.001
**Hallucinations**	61 (8.2)	10 (5.1)	2 (2.8)	8 (20.0)	8 (10.5)	89 (7.9)	0.009
**Agitation**	208 (27.8)	47 (24.0)	17 (23.9)	24 (60.0)	18 (23.7)	314 (27.8)	< 0.001
**Psychosis**	98 (13.1)	17 (8.7)	7 (9.9)	17 (42.5)	7 (9.2)	146 (12.9)	< 0.001
**Seizures**	13 (1.7)	1 (0.5)	2 (2.8)	1 (2.5)	3 (3.9)	20 (1.8)	0.18
**Palpitations**	22 (2.9)	32 (16.3)	5 (7.0)	3 (7.5)	7 (9.2)	69 (6.1)	< 0.001
**Chest pain**	35 (4.7)	40 (20.4)	1 (1.4)	1 (2.5)	7 (9.2)	84 (7.4)	< 0.001
**Arrhythmias**	1 (0.1)	-	-	-	-	1 (0.1)	1.00
**Total**	748 (100)	196 (100)	71 (100)	40 (100)	76 (100)	1131 (100)	

*GCS* Glasgow Coma Scale, *HR* Heart rate, *MDMA* Methylenedioxymethamphetamine, *RR* Respiratory rate, *SBP* Systolic blood pressure, *tp* temperature

^a^At presentation

^b^Missing: 4 (amphetamine 2, other 2)

**Table 4:** Clinical features of poisoning involving central stimulant drugs – hospital ED patients

	**Amphetamine *n* (%)**	**Cocaine *n* (%)**	**MDMA *n* (%)**	**Multiple *n* (%)**	**Total *n* (%)**	***p* value**
**Cardiac arrest**^a^	1 (0.8)	1 (3.1)	-	3 (6.0)	5 (2.4)	0.20
**Tachypnoea** *(RR* ≥ *20/min)*^a^	33 (27.3)	11 (34.4)	3 (37.5)	20 (40.0)	67 (31.8)	0.41
**Bradypnoea** *(RR* < *10/min)*^a^	9 (7.4)	1 (3.1)	-	4 (8.0)	14 (6.6)	0.88
**Tachycardia** *(HR* ≥ *100/min)*^a^	30 (24.8)	17 (53.1)	5 (62.5)	17 (34.0)	69 (32.7)	0.005
**Bradycardia** *(HR* < *50/min)*^a^	12 (9.9)	1 (3.1)	1 (12.5)	3 (6.0)	17 (8.1)	0.46
**Hypertension** *(SBP* ≥ *180 mmHg)*	11 (9.1)	10 (31.3)	4 (50.0)	7 (14.0)	32 (15.2)	0.001
**Hypotension** *(SBP* ≤ *90 mmHg)*	19 (15.7)	4 (12.5)	1 (12.5)	8 (16.0)	32 (15.2)	0.97
**Hyperthermia** *(tp* ≥ *39.0 °C)*	8 (6.6)	2 (6.3)	-	1 (2.0)	11 (5.2)	0.68
**Level of consciousness**^a,b^						0.035
*GCS score 15*	31 (26.5)	16 (50.0)	3 (37.5)	11 (22.9)	61 (29.8)	
*GCS score 8–14*	43 (36.8)	10 (31.3)	5 (62.5)	18 (37.5)	76 (37.1)	
*GCS score* ≤ *7*	43 (36.8)	5 (18.8)	-	19 (39.6)	68 (33.2)	
**Vomiting**	8 (6.6)	5 (15.6)	-	8 (16.0)	21 (10.0)	0.15
**Headache**	11 (9.1)	5 (15.6)	-	2 (4.0)	18 (8.5)	0.29
**Anxiety**	66 (54.5)	22 (68.8)	6 (75.0)	31 (62.0)	125 (59.2)	0.37
**Hallucinations**	15 (12.4)	8 (25.0)	4 (50.0)	11 (22.0)	38 (18.0)	0.021
**Agitation**	51 (42.1)	12 (37.5)	4 (50.0)	25 (50.0)	92 (43.6)	0.66
**Psychosis**	10 (8.3)	5 (15.6)	-	6 (12.0)	21 (10.0)	0.51
**Seizures**	12 (9.9)	3 (9.4)	1 (12.5)	6 (12.0)	22 (10.4)	0.92
**Palpitations**	4 (3.3)	4 (12.5)	-	2 (4.0)	10 (4.7)	0.21
**Chest pain**	5 (4.1)	7 (21.9)	-	4 (8.0)	16 (7.6)	0.016
**Arrhythmias**	5 (4.1)	8 (25.0)	-	5 (10.0)	18 (8.5)	0.005
**Hyperlactataemia** *(*≥ *3.0 mmol/L)*^*a*^	9 (7.4)	9 (28.1)	1 (12.5)	9 (18.0)	28 (13.3)	0.011
**Total**	121 (100)	32 (100)	8 (100)	50 (100)	211 (100)	

*ED* Emergency department, *GCS* Glasgow Coma Scale, *HR* Heart rate, *MDMA* Methylenedioxymethamphetamine, *RR* respiratory rate, *SBP* Systolic blood pressure, *tp* temperature

^a^At presentation

^b^Missing: 6 (amphetamine 4, multiple 2)

At the outpatient clinic, agitation, psychosis, and hallucinations were most prominent in the other stimulants group, and 24/40 (60.0%) of the patients in this group were transferred to hospital (Tables 1 and 3). The other stimulants group also encompassed patients having taken unspecified stimulants, and among these 18/23 (78.3%) were agitated, 12/23 (52.2%) were psychotic, and 17/23 (73.9%) were transferred to hospital.

## Discussion

### Summary of main findings

Amphetamine was involved in 73% of cases with central stimulant drug poisoning, cocaine in 23%, and MDMA in 11%. Amphetamine was often combined with opioids and benzodiazepines. Cocaine and MDMA were mainly combined with ethanol and often occurred during weekends. Cardiotoxic effects were more frequently seen when cocaine was involved. Agitation and psychosis were particularly widespread among patients having taken unspecified stimulants. Most patients presenting to the primary care emergency outpatient clinic were managed at this level, but 17% were transferred to a hospital for medical review and 8% to a psychiatric hospital. Among the hospital patients, 93% were admitted to intensive care.

### Comparisons between drugs

Amphetamine was frequently combined with opioids. This explains why naloxone frequently was given in the amphetamine group and likely signifies that an opioid toxidrome often dominated the clinical picture of the combination. Most of these patients were probably injecting drugs, as an estimated 3000–4000 people in Oslo regularly do [13]. In a Norwegian study of people injecting drugs, 60% had injected amphetamine during the last 4 weeks, 43% had injected heroin, and 29% both [14]. In our study, many patients combining amphetamine and opioids had also taken benzodiazepines, signifying dangerous polydrug use in this group of patients [15, 16]. Most drug-induced deaths in Oslo are polydrug poisonings [17], and fatal overdoses from combined stimulant and opioid poisoning are on the rise in the USA [2].

Patients taking cocaine and MDMA were significantly younger than those taking amphetamine, probably reflecting the position of these drugs as party drugs or club drugs [18, 19]. This is consistent with findings across Europe [20]. Along the same lines, ethanol co-ingestion was most frequent among cocaine and MDMA patients, and they more frequently presented on weekends. GHB, often also viewed as a party drug [18, 21] and previously found to have a weekend presentation pattern in Oslo [22], was surprisingly often combined with amphetamine.

Taking multiple stimulants seemed to be a major risk factor for severe toxicity. The largest proportions needing intubation and sedation at the hospital were found in this group. However, the patients in the cocaine group stayed the longest in the hospital. Chest pain, tachycardia, and arrhythmias were mostly seen when cocaine was taken, in line with the established cardiotoxic effects of cocaine [23].

Psychosis is a known risk of amphetamine use [24, 25]. Although most of the patients presenting with psychosis had taken amphetamine, the largest proportion with psychosis and agitation was seen among patients taking unspecified stimulants. The unspecified stimulants may hide undiagnosed stimulant NPS inducing psychosis [26, 27], though it is also possible that these stimulants were registered as unspecified as the patients were too psychotic to give any specific information on the drugs taken.

Nearly all the hospital patients were admitted to intensive care. Hence, triage for hospital treatment seems to have been appropriately targeted by the ambulance service and the outpatient clinic. Concerning the related risk of under-triage, a previous study found that patients with substance use-related poisoning were safely managed in primary care by the procedure in use at the OAEOC [11].

### Time trends

Compared to a study from 2012 at the OAEOC [22], there was a 17% increase in the number of amphetamine poisonings per year, while the number of cocaine and MDMA poisonings per year had doubled. A similar trend is seen in the USA, where both fatal and non-fatal overdoses from stimulant poisoning are increasing [2]. The increase in MDMA poisonings matches the increase in MDMA seized by Norwegian police since 2010 [3]. Surprisingly, no corresponding change in the seizure of cocaine has been seen during the last decade [3]. In wastewater analyses, gradually increasing amounts have been found since 2010 for all three drugs: amphetamine, cocaine, and MDMA [4]. Though we found an increasing number of cocaine poisonings, figures are still low compared to other ED settings in Europe [28].

### Strengths and limitations

We included patients both at the OAEOC, where most substance use-related poisonings in Oslo are treated [11], and at one of the four Oslo hospitals. By including the hospital level, where the more severe cases are treated, our results should be representative for the Oslo area. We did not include patients left on the scene after treatment by the ambulance service, but these poisonings mainly involve opioids and ethanol [29].

The diagnosis of toxic agents was registered from the patient records and mainly based on the clinical assessment made by the doctor treating the patient, in turn mainly based on information from the patient and companions. Laboratory confirmation was not done at the outpatient clinic and only in 60% of the hospital patients, leading to an uncertainty in the diagnosis of toxic agents. This may also have led to an underreporting of infrequently occurring substances. However, a previous Oslo study with toxicological testing for a broad range of psychoactive substances found that patients usually had taken what they reported [27]. Still, 8% had taken an NPS not reported by the patient or clinically suspected by the doctor [27].

As we did not gather any follow-up data, we do not know whether any patients died shortly after discharge. This is especially a concern at the outpatient clinic where the median length of stay was as short as three hours. However, in previous studies of acute poisoning in the same setting, death shortly after discharge was extremely rare [11, 29, 30].

We co-categorized amphetamine and methamphetamine. Drug users in Norway rarely distinguish between them [3]. Norwegian police and customs seizures of methamphetamine increased after 2000 and have been in the same range as amphetamine during the last decade [3].

## Conclusions

Amphetamine was the most common central stimulant drug involved in acute poisoning in Oslo, often combined with opioids and benzodiazepines among people injecting drugs, constituting severe polydrug poisonings in an at-risk population. Cocaine and MDMA were often combined with ethanol and were more frequently seen during weekends, reflecting party and club use. Cardiotoxicity was more frequent when cocaine was involved. The most severe toxicity was seen when multiple stimulant drugs had been taken in combination. The cases involving unspecified stimulants had more severe psychiatric symptoms. Future research should use toxicological laboratory testing to identify the specific stimulants involved.

## Data Availability

Data are currently not available for sharing. Several manuscripts based on the data set are in preparation. Requests concerning the data may be sent to the corresponding author.

## Abbreviations

ED: Emergency department
GC-FID: Gas chromatography–flame ionization detector
GC-MS: Gas chromatography–mass spectrometry
GCS: Glasgow Coma Scale
GHB: Gamma-hydroxybutyrate
HR: Heart rate
LC–MS/MS: Liquid chromatography–tandem mass spectrometry
LSD: Lysergic acid diethylamide
MDMA: Methylenedioxymethamphetamine
NPS: Novel psychoactive substances
OAEOC: The Oslo Accident and Emergency Outpatient Clinic
OUH: Oslo University Hospital
PMMA: Paramethoxymethamphetamine
RR: Respiratory rate
SBP: Systolic blood pressure
tp: Temperature
